# Dynamic Plus-Delta: an agile debriefing approach centred around variable participant, faculty and contextual factors

**DOI:** 10.1186/s41077-021-00185-x

**Published:** 2021-10-07

**Authors:** Ranjev Kainth

**Affiliations:** 1 Faculty of Life Sciences and Medicine, King’s College London, London, UK; 2 Simulation and Interactive Learning (SaIL) Centre, Guy’s and St Thomas’ NHS Foundation Trust, London, UK

**Keywords:** Dynamic, Plus-Delta, Debriefing, Mass-scale simulation, Nightingale, Psychological safety, Psychological well-being, Novice faculty

## Abstract

The current coronavirus pandemic has necessitated rapid intensive care infrastructure expansion with corresponding demand for training healthcare staff. At the NHS Nightingale Hospital, London, the staff underwent a training programme prior to entering the clinical environment with simulation being a core component. This paper describes the rationale for choosing an initial debriefing model which evolved overtime to consider multiple contextual factors: demands of the clinical environment, the diverse participants and their learning needs, the variable experience of faculty, and the dynamic nature of available debriefing time. The new approach, termed here as the Dynamic Plus-Delta model, blends the traditional Plus-Delta approach with specific dynamic elements which considers the unique demands of rapidly training large number of staff. We outline the core features of this model and detail specific considerations around psychological safety. This debriefing approach can be used in similar simulation intervention settings where rapid training of participants is required with multiple and varying contextual factors.

The ongoing pandemic has necessitated new ways of working within the healthcare sector including novel methods to deliver healthcare services. An important component when considering new services is the pre-implementation phase where the focus is on aspects such as the training of healthcare professionals. This paper details the evolution of a debriefing model in the context of needing to rapidly train large numbers of healthcare professionals in a simulated intensive care environment. Issues around faculty experience, different learner groups and local factors such as time and changing clinical demand influenced the advancement of the traditional Plus-Delta approach to what is labelled here as a Dynamic Plus-Delta model. The model is discussed alongside the methods employed to foster participant and faculty psychological safety which are critical to success in the context of the pandemic and with such variable contextual factors. This model can be adapted and implemented by other educators depending on their local context and needs.

## Background: the need for an educational intervention

As we rapidly approach the second year anniversary since the World Health Organization declared a ‘public health emergency of international concern’ [1], numerous countries across Europe and the world continue to experience a resurgence of novel coronavirus infections with corresponding demands on healthcare services [2–4]. The initial outbreak placed a significant strain upon health systems in China resulting in the rapid expansion of intensive care facilities, construction of dedicated field hospitals and conversion of public venues to care for patients [5, 6]. Similar demands were seen across the world with the construction of new dedicated healthcare facilities and emergency planning in places such as France [7], New York [8] and Italy [9]. In England, dedicated field hospitals (National Health Service (NHS) Nightingale Hospitals) were constructed at three locations with the largest built in London [10, 11] in anticipation of a surge in intensive care demand. In parallel, individual hospitals increased their in-house capacity, with expansion continuing in preparation for subsequent waves. Alongside rapid infrastructure expansion, there was a necessary personnel expansion and associated training, particularly when non-intensive care staff were redeployed to manage the surge in patient numbers [12].

At the beginning of the pandemic in England, a dedicated training facility was commandeered, initially at the site of the NHS Nightingale Hospital, London, and later at The O2, a large entertainment arena, with over 2500 individuals completing the programme. Simulation has been placed at the centre of preparation and training during the pandemic across the globe [13, 14] and was an integral feature of the training programme for staff entering the NHS Nightingale Hospital in London.

## The NHS Nightingale Hospital: the simulation station

The delivery of education for staff entering the NHS Nightingale Hospital, London, consisted of different stations and workshops, each focused on specific content related to clinical practice. For example, there were stations on personal protective equipment (PPE), communication and hand signals and using blood gas machines. There was a specific simulation station which consisted of a simulated intensive care bed, set up in the same way as a typical NHS Nightingale Hospital bed. In total, eight simulated beds were constructed each with a separate debriefing area enabling many participants to be actively involved simultaneously.

The simulation station consisted of a briefing phase, one or two simulated activities and a corresponding number of debriefs. Participants tended to be grouped together based upon similar backgrounds and competence levels although it was common to have intra-group variance. Specifically, the training evolved to have four main groups with each allocated different time in the simulation station: ‘green’ group consisting of experienced intensive care staff, ‘amber’ group consisting of non-intensive care medical and nursing staff, ‘red’ group consisting of non-healthcare individuals, and ‘purple’ group consisting of pharmacists. Colours were used on name badges to identify the group members and on the daily programme so the faculty could plan for different groups in advance.

## A rapidly changing context

Some training days encompassed over 200 participants, and multiple different coloured groups meaning faculty had to rapidly adjust to new learners, objectives and session timings. It was not unusual to move between facilitating experienced intensive care staff followed immediately to non-healthcare administrative staff and similarly between small participant numbers where only two simulated beds were required to sessions where four or more were needed to maintain physical distancing.

In addition to this, the learning objectives continually evolved and adapted contemporaneously to clinical demand and changed in response to learners on the day. Participant groups, numbers and learning objectives all influenced the available time for debriefing which was further confounded by overall scheduling for the training day which could change at short notice due to logistical issues.

Similar challenges will also exist when training is undertaken within hospitals expanding their intensive care facilities with the added complexities associated with in-hospital and in-situ simulation. Whilst certain content is fixed, such as standardised coronavirus-specific communication (e.g. hand signals, bedside checklists) and protocols (e.g. intubation, cardiac arrest), this may not be relevant to all participants, particularly as there is a spectrum of learners ranging from non-healthcare participants to senior intensive care participants with a corresponding shift in learning needs and required training [15, 16]. As such, the chosen debriefing model needed to account for all these abovementioned contextual factors whilst equally catering for a diverse faculty mix.

## Debrief model requirements: satisfying a diverse faculty mix

There is substantial and accepted evidence that debriefing is a central component in the learning process for healthcare participants [17–19]. Much of debriefing is centred around models with different phases and specific conversational structures [20]. Whilst many authors recognise the complexity in debriefing and claim the models remain accessible to novice debriefers [21, 22], many of the approaches require previous experience in debriefing and often a dedicated course as a pre-requisite to practise [23]. In addition to managing an evolving debrief with the challenges of co-facilitation [24], the faculty must foster and sustain psychological safety amongst participants [25], further increasing their own cognitive load [26].

The pandemic is a specific example of a situation where the speed of education delivery is paramount and, in such situations, faculty are recruited firstly upon availability rather than expertise. This is true both at mass-scale simulation venues and hospitals endeavouring to expand in-house services [12]. This inevitably leads to a mixture of skilled and inexperienced educators and is a reflection on the challenges the simulation community faces with access to high-skilled faculty [27]. At the NHS Nightingale Hospital, London, the simulation teaching faculty was more than 30 individuals (‘core group’) from diverse backgrounds including intensive care staff, paediatric nurses and doctors, paramedics, anaesthetists and internal medicine doctors with clinical experience ranging from two years to several decades. Within this group, there were some educators experienced in debriefing including full-time simulation educators and others who had only taught in clinical environments with no training regarding specific debriefing models. As a result, there was no common debrief model across the entire faculty. In addition to these educators, there was a second group who were involved sporadically with the simulation team with the rest of their time spent in different non-simulation-based education stations.

Typically, training days involved around 15 teaching simulation faculty. Whilst the core group became familiar with one another over time, many had never met one another prior to the teaching days, and individuals joined the core team at different times introducing a further variable which is not typical of centre-based simulation or established educational programmes. In addition to this, the faculty from the second group would often join the core group unannounced with no previous experience teaching on the station.

All educators underwent generic induction training to the Nightingale education team which ensured faculty were uniformly aware of the demands and expectations of being part of the simulation faculty. However, the complex logistics of the entire training programme meant it was not feasible, nor appropriate, to train educators to use a uniform debrief model, especially as standardised courses can run for several days. The debrief choice must therefore satisfy the competence and confidence of the available faculty without the need for dedicated training: an off-the-shelf approach which any healthcare professional could reasonably be expected to execute.

Further, the debrief choice is shaped by other factors including the dynamic nature of available time, the variance of learners, and ensuring the specific learning objectives are met [28]. In this specific setting, the focus of debriefing is not primarily on team behaviours or individual cognitive frames. Rather the function of mass-scale simulation is to prepare individuals for the specific environment, protocols and procedures related to coronavirus positive patients in intensive care—a systems approach to improving patient safety whilst simultaneously maintaining the safety of healthcare professionals entering a highly infectious environment. A similar approach is also required in other mass-scale simulation settings such as natural disasters or war. Success in debriefing here therefore means placing system issues at the centre of discussions, particularly as many of the protocols and procedures may be new during the pandemic and likely to cause confusion and anxiety [13].

## Plus-Delta: traditional approach and questioning style

The chosen debrief model we initially employed at the Nightingale training programme was the traditional Plus-Delta approach which is already used extensively in simulation-based education [28, 29]. We were confident faculty would be familiar with the traditional approach and those who were new to debriefing were likely to recognise the questions from other setting such as undergraduate nursing and medical education [30, 31].

**Fig. 1:**
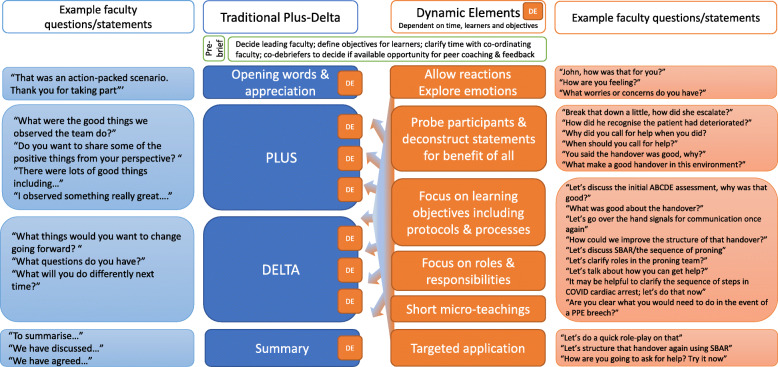
The Dynamic Plus-Delta model. The Dynamic Plus-Delta model combines the core phases of the traditional Plus-Delta model with dynamic elements which can be introduced throughout and at any time during the debriefing. Example phrases are provided which demonstrate how the model is responsive to the demands of the situation

Plus-Delta has often been described as either a questioning approach integrated within other debrief models (such as PEARLS [22] and TeamGAINS [23]) or as a stand-alone model as described by Motola et al. (2013), Fanning and Gaba (2007) and Oriot and Alinier (2018) [28, 29, 32]. Irrespective of how the concept of Plus-Delta is implemented, the core feature is the focus on participant self-assessment detailing positive aspects of the experience (the ‘plus’) and things to change (the ‘delta’). The first column in Fig. 1 details how each of these traditional phases can be executed with example faculty discourse. The facilitator and observers often add to the list of plus/delta responses to further knowledge sharing, and the reflections surfaced may be at individual, team or system-wide issues [28].

Recent evidence shows that modified versions of the traditional approach are being used in debriefs after in-hospital cardiac arrest to improve patient care [33]. In this study by Wolfe et al. [33], the two core phases were combined with details regarding the clinical case, case events and cardio-pulmonary resuscitation quality (Wolfe, H. written communication, May 22, 2021), demonstrating the utility of combining core Plus-Delta phases with elements related to relevant contextual demands.

The Plus-Delta model was utilised in the Nightingale training programme and was specifically chosen as it does not necessitate specialised training and was familiar amongst the faculty. However, due to needing to readily adapt to changes in context, learners and learning needs, a modified version evolved over time which is labelled in this paper as the Dynamic Plus-Delta approach.

## Dynamic Plus-Delta: an evolved approach

The rapidly changing context and the variable length of debriefing time necessitated new elements to be introduced into the traditional model to meet the needs of the educational programme. Debriefing thus took the shape of a Dynamic Plus-Delta model: flexible and adaptable to the immediate context which changed frequently throughout the day.

The core components of the Dynamic Plus-Delta approach are outlined in Fig. 1 and consists of three main phases: the opening phase, the plus-delta phase where most time will be spent and a summary phase where targeted application takes place. Where more time is available, there is a greater degree of flexibility and with less time, specific elements are chosen dependent upon the target group.

Rather than entering directly into the positive assessment of performance as is the case with the traditional Plus-Delta model, there is an option to commence with the exploration of emotions and reactions. In shorter debriefs with experienced participants, less time may be spent in this phase; the decision is based upon local need and is thus not a fixed element.

The debrief moves to the plus-delta phase and is an opportunity to introduce further relevant dynamic elements. Dynamic elements are outlined in column 3 in Fig. 1 and are in essence different strategies for faculty to employ depending on the immediate context and available time. Short descriptions of the dynamic elements are presented in Table 1.

**Table 1:** Dynamic elements. Dynamic elements are different strategies which can be introduced throughout the debrief in different phases and in combination with one another. Time and learner needs will dictate which elements receive greater emphasis

Dynamic element	Phase	Description
Allow reactions Explore emotions	Opening Plus-Delta	Initial reactions and emotions will be related to the simulation scenario, the simulated bed space, entering the clinical environment, and related to the pandemic and may surface during different phases. Emotions will differ for those experienced in intensive care settings compared to participants who may have never worked in healthcare. When time permits, it is important to normalise and explore emotions as other participants in the group are likely to share similar emotions.
Probe participants and deconstruct statements for benefit of all	Plus-Delta	To facilitate shared learning, a deeper exploration of the underlying thought processes and perspectives is required. This is vital both when there is a positive assessment provided and if there are suggestions for change. This is a strategy which may be used if participants begin to discuss learning objectives or group-specific content.
Focus on learning objectives including protocols and processes	Plus-Delta	There are likely to be specific learning objectives related to the clinical context including new protocols and processes. These can be discussed in detail as they arise, or faculty may choose to specifically raise these as topics for discussion. Some content may be delivered in short microteachings, particularly when time is limited.
Focus on roles and responsibilities	Plus-Delta	Different coloured groups will have different roles and responsibilities, and time can be spent clarifying what these are for participants and the expected roles and responsibilities of other staff in the clinical environment.
Short microteachings	Plus-Delta	These may be used to recap the standardised information from previous sessions or deliver group-specific content. This element can also be introduced if there is a need to cover core learning objectives or group-specific information, especially if time is limited.
Targeted application	Summary	An opportunity for participants to conceptualise working in the clinical environment by practising new knowledge or concepts or to re-try actions from the scenario. Examples include getting participants to structure a SBAR handover, calling for help or envisaging how protocols would logistically be executed in the clinical environment.

The function of these elements is to ensure critical group and context-specific objectives are met. For example, the element ‘short microteachings’ can be used to recap standardised information from previous sessions in the day and would be applicable to all groups and may become a ‘standing item’. For the red and amber groups, there may be a focus on objectives centred around the ABCDE assessment (Airway, Breathing, Circulation, Disability, Exposure) of the ventilated patient, and more time may be needed in exploring emotions such as fears of working in the intensive care environment. In comparison, the green groups may wish to spend more time clarifying new protocols and associated roles, for example, around intubation and in such situations where time is limited, it may be appropriate for the faculty to combine these discussions with elements of directive feedback, for example, centred around system issues. In this way, the debrief is tailored and adapted to the learners’ needs in each group.

These elements are built into the plus and delta phases rather than ‘add-ons’. For example, if comments around ‘calling for help’ surfaced during the positive phase, it is an opportunity to deconstruct the underlying intentions or the ‘why’ behind the positives which Dieckmann et al. [34] argue is a strategy to enable ‘learning from success’—an approach which may be missing from the traditional approach [32]. This allows other participants to understand the underlying rationale behind positive action and behaviours and would be more relevant to some learners (for example, the red group). Further education, if required, around calling for help can then be delivered by targeted microteachings. Through this approach and introducing various dynamic elements, group-specific learning objectives can be met.

There may be opportunities during the closing summary phase for participants to practise new knowledge or concepts or to re-try actions from the scenario such as a handover. For example, rather than simply stating that handover should be delivered in a SBAR structure (situation, background, assessment, recommendation), participants are collectively encouraged to construct sentences—a form of a targeted application. As a result, the clinical environment will not be where participants practise this new knowledge. This active experimentation, as part of Kolb’s experiential learning cycle, exhibits the ethos that ‘learning is an emergent process’ rather than an outcome (page 26) [35]. The process of practising skills in the final debrief phase is also seen in the TeamGAINS model (step 6) [23].

The Dynamic Plus-Delta approach is similar to the PEARLS for Systems Integration method (PSI) [36] which includes conversational techniques such as circular questioning [23] and advocacy and inquiry [37] during the main discussion and the depth adjusted based on available time. The PSI is centred around systems issues and addressing stakeholder needs and does not directly consider the dynamic nature of learners and learning objectives. Further, we intentionally centred the debriefing approach around the traditional Plus-Delta recognising the diverse experience of faculty and the prospect that other questioning approaches require both a higher level of faculty expertise and more time to foster higher levels of psychological safety when depicting specific participant actions. In addition, when more time was available, rather than explore cognitive frames or team dynamics through these methods, we instead dissect the experience in relation to system-related and process-driven issues [32, 38]; the facilitator is able to explore in depth, select topics raised with a focus on solution and improvement seeking from other faculty and participants [32, 39].

The traditional Plus-Delta debrief approach has previously been used to examine some of the processes, checklists and protocols imagined in the neo-clinical environment [40, 41] and highlighted the changes and clarification required from and to the clinical environment. Previous research also suggests this method enhances the learning experience of participants in specific process-related simulations [42] and thus relevant for staff entering the NHS Nightingale Hospital, London. In addition to being flexible to adapt to learning needs, the model also considered the psychological well-being of staff with a strong emphasis to create psychological safety to enable dialogue around participants’ emotions: prudent in the context of a pandemic.

## Understanding, fostering and sustaining psychological safety

Fostering and maintaining psychological safety is key for successful debriefing, particularly with such diverse participants and faculty and in situations of rapid simulation intervention implementation. It is critical to enable participants to speak up, ask questions, seek clarification and feedback, and report potential problems [37, 43]. This is pertinent in the current climate as many protocols and processes will be new, and it is vital to both staff and patient safety that participants have a clear understanding of working practices and are empowered to flag up potential problems. The strategies outlined below are a result of iterative work by the simulation team over time and will be equally valid in local settings where single as opposed to multiple concurrent simulations are conducted. These strategies should be used in conjunction with recognised published work in the field such as principles for constructing a safe container by Rudolph et al. [44] and the implicit and explicit strategies outlined by Kolbe et al. [25].

## Keeping participants safe

Psychological safety was fostered through explicit strategies such as small group introductions and demonstrating care for the individual through maintaining physical distancing and providing sanitising hand gel. To maximise physical distancing, participants were split into smaller groups, and as a result, up to eight simulations and debriefs would run concurrently and is the prime reason for a large faculty pool. The beginning of each session included introductions to explore participants’ background and concerns, recognising the baseline psychological safety of participants differ and, if not addressed, can impact engagement in the ongoing or the upcoming activity [45, 46]. This will be particularly relevant when training teams or entire wards at hospitals conducting in-house training. Standard practice was to have two facilitators per group with one leading the debrief. Participants were arranged in circles in line with other coronavirus simulation debriefing approaches [13] with faculty seated amongst them to further foster psychological safety [47].

In addition to some overt strategies, debriefers also considered questioning strategies. Rather than analysing individual performance and their cognitive frames, which requires more significant work in terms of fostering psychological safety and being skilled in specific conversational approaches, the focus was to highlight good performance, focus discussion around areas for improvement, and relating the simulation to the neo-clinical environment. Such an approach also minimises the need to write detailed notes as suggested in other debrief models as instead, macro moments can be recalled by participants. During discussions, faculty focused on small group strategies such as respectful listening, equal airtime and adopt a debrief stance demonstrating honesty, genuine curiosity and hold the learner in positive regard, pertinent in view of the wide range of participant competence and experience [48, 49].

As faculty backgrounds were diverse, with many never having worked in adult intensive care, we emphasised the importance of an open and honest approach from faculty who shared their own uncertainty regarding clinical practice, protocols and systems setup in the new environment. Such candidness, defined by Walters and Diab (2016) as ‘humility’ or ‘humble leadership’, was important primarily for patient safety and also helped faculty connect with participants, further maintaining a psychologically safe environment [50].

In parallel, faculty also recognised emotions of participants, many of whom may never have experienced intensive care and a substantial proportion never having worked in healthcare. From our experience, it was during the simulations and seeing a typical intensive care bed with a ventilated patient when participants began to conceptualise how the different sessions in induction would coherently come together; this often led to emotions of uncertainty, fear and anxiety. Anticipation (at pre-simulation briefings—see below), recognition, appreciation and normalising such emotions were therefore a key part of the debrief. The act of debriefing together and sharing emotions fosters team cohesion and empathy [29] which is linked to patient safety [51]. Sharing of immediate emotions in relation to the simulations occurred at the start of debriefing; however, it was also a dynamic element, which meant faculty were empowered to examine emotions throughout the session.

## Proactively managing faculty psychological safety

The concept of faculty psychological safety has not been extensively explored in the simulation literature, and the training at the Nightingale Hospital, London, created a situation where this concept was proactively managed before, during and after the daily simulations.

The faculty briefing and debriefing was led by a non-debriefing co-ordinating facilitator who received information regarding participant numbers, group colours and timings for the day. At pre-simulation faculty briefings, there was a standardised sequence of events: full faculty introductions so new members felt part of the team, discussion of concerns around aspects such as equipment or logistical issues, clarification of clinical (content) knowledge and expectations of faculty (pairings, debrief leads and coaching) and of participants (who to expect and how we would manage). When simulation sessions were spread across the day, there would be a second, shorter briefing prior to starting the afternoon session allowing multiple opportunities to come together as a group and to discuss issues. During the end of the day faculty meeting, there was a focus on faculty experience, led through narrative discourse rather than a structured approach. This included technical issues, general questions and feedback, and on occasions, this would lead to short learning conversations.

During the debriefs, junior members of the team (defined as either being new to the group or novice debriefers) were paired with more experienced faculty. This also allowed peer coaching between some of the senior faculty members with junior members creating a culture of continued learning within a psychologically safe environment [52] and enabling novice faculty to observe the expected teaching routine and standards. We employed a ‘follow the leader’ approach as outlined by Cheng et al. [24] to ensure structured co-debriefing. When several groups were running concurrently, the faculty co-ordinator would float between the debriefs which were running in parallel. This was primarily to act as an additional knowledge source when questions would arise during the debrief which the debriefing faculty were unable to provide an answer, often because of the rapid changes in the clinical environment. Having access to an additional knowledge source further helped to reduce the cognitive load on novice faculty.

Collectively, the multiple group meetings, consistent introductions, opportunities to raise concerns and nurtured approach through co-debriefing and a lead co-ordinator facilitated the construction of a safe environment for faculty. It became apparent that these were core principles in forming a learning culture which would be equally applicable to other settings where unfamiliar faculty from diverse backgrounds come together in new situations.

## Conclusion

The Dynamic-Plus-Delta model was born out of the current pandemic where an agile, yet accessible approach was required to fulfil the needs of mass-scale simulation. This approach is suitable in situations where aspects such as participant background, learning objectives, faculty skill and available time are variable. Whilst no formal evaluation of the model was conducted, there is a degree of face validity considering over 2500 participants experienced the model and feedback from participants, and clinical teams working inside the hospital centred on adjusting content (which the model is designed to accommodate for) rather than the format of the model.

The approach will be particularly suitable in situations of in situ simulation for new staff, new environments, and testing new processes and protocols. The approach is flexible enough to allow local teams to adapt the model and introduce new dynamic elements or ensure certain elements such as microteachings are standing items within the debrief when there is a defined and fixed learning need. The key to success here, as with our experience at the NHS Nightingale Hospital, London, is the engagement of faculty and understanding and establishing psychological safety to enable safety-related discussions to emerge.

As we enter new phases of the pandemic, including the distribution of vaccines, simulation training will continue to play a central role in training healthcare professionals [53]. The Dynamic Plus-Delta model is an approach which developed from a traditional model and remains accessible to faculty with variable expertise and can be operationalised in multiple different and rapidly changing contexts.

## Data Availability

No additional data and materials are available.

## Abbreviations

DAA: Description, analysis, application
NHS: National Health Service
ABCDE: Airway, Breathing, Circulation, Disability, Exposure
PSI: PEARLS for Systems Integration
SBAR: Situation, Background, Assessment, Recommendation
DE: Dynamic element
